# Genetic and environment influences on childhood victimization: a systematic review and meta-analysis

**DOI:** 10.1038/s41380-024-02868-z

**Published:** 2024-12-11

**Authors:** Tarik Dahoun, Alicia Peel, Jessie Baldwin, Oonagh Coleman, Stephanie J. Lewis, Jasmin Wertz, Frühling Rijsdijk, Andrea Danese

**Affiliations:** 1 Department of Child and Adolescent Psychiatry, Institute of Psychiatry, Psychology and Neuroscience, King’s College London, London, UK; 2 Our Future Health, Manchester, UK; 3 Social, Genetic and Developmental Psychiatry Centre, Institute of Psychiatry, Psychology and Neuroscience, King’s College London, London, UK; 4 Department of Clinical, Educational and Health Psychology, Division of Psychology and Language Sciences, University College London, London, UK; 5 Department of Psychology, School of Philosophy, Psychology & Language Sciences, University of Edinburgh, Edinburgh, UK; 6 Psychology Department, Faculty of Social Sciences, Anton de Kom University of Suriname, Paramaribo, Suriname; 7 National and Specialist CAMHS Clinic for Trauma, Anxiety, and Depression, South London and Maudsley NHS Foundation Trust, London, UK

**Keywords:** Psychology, Genetics

## Abstract

Childhood victimization is a key risk factor for poor mental and physical health. In order to prevent childhood victimization, it is important to better understand its underlying etiological factors. Childhood victimization is not randomly distributed in the population but occurs more often in the context of certain characteristics of the child, the family, and the broader environment. These characteristics may be both genetically and environmentally influenced, making genetically informative designs valuable to disentangle the etiological factors. Here we performed meta-analyses of the genetic and environmental influences on childhood victimization based on twin studies. We also tested whether genetic and environmental influences on childhood victimization vary depending on key features of victimization experiences including the reporter of victimization experiences, the type of victimization, and the age at exposure. Following PRISMA guidelines, a search for relevant literature was conducted using MEDLINE, APA PsycInfo, and Embase databases until September 2023. A meta-analysis based on 21 studies with 62,794 participants showed that genetic influences accounted for 40% of the variance in childhood victimization, shared environmental influences for 20%, and non-shared environmental influences for 40%. In addition, we found that genetic and environmental influences on victimization varied based on the reporter and the type of victimization, and the age at victimization. The quantitative summary of genetic and environmental influences provided by this study advances our understanding of the mechanisms underlying risk for childhood victimization and points to prevention targets for victimization and its health effects.

## Introduction

Children who are maltreated by adults or bullied by peers are at greater risk of poor mental and physical health than non-victimized peers [1–7]. In order to prevent child victimization and mitigate its effects on health, it is essential to better understand the influences underlying its occurrence. Childhood victimization does not occur randomly in the population. Instead, it occurs more often in the context of certain characteristics of the child, the family, and the broader environment including cognitive difficulties [8], neurodevelopmental conditions [9–11], parental mental illness [12], and socio-economic disadvantage [13, 14].

Risk factors for childhood victimization consist of complex mixtures of genetic and environmental influences [15]. For example, risk factors for childhood victimization, such as intelligence, ADHD, and psychopathology, all show heritable components [15]. Because such risk factors are partly heritable, the risk for childhood victimization itself presumably emerges from a mixture of genetic and environmental influences. The intergenerational transmission of childhood maltreatment (i.e., higher risk of maltreatment in children of parents with a history of maltreatment) has also long been debated [16] and similarly highlights the relevance of gene-environment interplay in victimization risk. Several studies have investigated this question but reached inconsistent results, highlighting the need for a quantitative summary and a better understanding of the heterogeneity in the literature. Greater appreciation of the genetic and environmental influences on childhood victimization can provide novel insights into the underlying risk processes and help identify modifiable targets for interventions. Of note, evidence of genetic influences does not equate to deterministic effects. A classic, reductionist example is phenylketonuria--a monogenic disorder that is 100% heritable but whose clinical manifestations can be entirely prevented with targeted dietary interventions [17]. Childhood victimization, of course, emerges through far more complex bio-psycho-social processes, but evidence of genetic (as well as environmental) influences might equally help disentangle risk mechanisms. Similar premises have promoted the adoption of genetic methods in other areas of social science research [18].

To explain the heterogeneity of the findings in the literature, we consider that the relative contribution of genetic and environmental influences on child victimization might differ based on the reporter or type of, or age at exposure to, victimization. Regarding the reporter of victimization, retrospective self-reports capturing the first-person perspective of affected individuals and prospective measures typically based on informant reports identify different groups of individuals [19] and might be influenced by different characteristics. For example, self-reported measures of environmental experiences show greater heritability than experiences assessed by direct observation [20], likely due to genetic influences on traits affecting appraisal and reporting (e.g., neuroticism [21], and sensory processing sensitivity [22]). Regarding the type of victimization, experiences that occur outside the family, such as bullying victimization by peers, might be less influenced by the family environment compared with maltreatment, which more often occurs at home. Regarding the age at exposure, young people’s genetic characteristics may have a stronger influence on victimization in adolescence than in childhood, as adolescents have more independence to select and explore their environment outside the family.

To expand our understanding of the influences on childhood and adolescent victimization, we have undertaken meta-analyses of the underlying genetic and environmental influences based on twin studies. Although the gene-environment interplay underlying childhood victimization risk has been more recently examined using alternative study designs (e.g., adoption studies, extended family design studies, and genome-wide association studies), the classical twin design [23, 24] has been the staple of investigations in the area and it will be the focus of this review. We have also tested whether genetic and environmental influences on childhood victimization vary depending on key features of victimization experiences.

## Methods

### Protocol and registration

This meta-analysis was pre-registered in the PROSPERO International Prospective Register of Systematic Reviews (https://www.crd.york.ac.uk/prospero/display_record.php?ID=CRD42021295222). Deviations from the pre-registered protocol are discussed in the Supplementary materials. We conducted this meta-analysis in line with the PRISMA guidelines [25].

### Inclusion criteria

We included original, peer-reviewed full articles written in English with the following criteria: (1) twin studies reporting monozygotic (MZ) and dizygotic (DZ) twin correlations for child victimization; (2) measures of child victimization defined as physical abuse, sexual abuse, emotional abuse, physical neglect, emotional neglect, and/or bullying occurring before age 18 years.

### Literature search

We searched MEDLINE, APA PsycInfo, and Embase databases for peer-reviewed articles written in English and published from inception (1946, 1806, and 1974, respectively) to August 31st, 2023, that included estimates of heritability of child victimization from twin studies. We used the following search terms: (“child*“ OR “young people” OR “young person” OR “youth” OR “adolescent*“ OR “pediatric” OR “juvenile”) AND (“advers*“ OR “early life stress” OR “stressful life event” OR “adverse childhood experiences” OR “maltreatment” OR “victimization” OR “ACE” OR “maltreat*“ OR “trauma” OR “abuse” OR “physical abuse” OR “emotional abuse” OR “psychological abuse” OR “sexual abuse” OR “neglect” OR “molest*“ OR “bullied” OR “bully”) AND (“twin*“ OR “monozygotic” OR “dizygotic” OR “heritability” OR “adopt*“ OR “family study” OR “gene-environment correlation” OR “rGE”). We also screened the references of included articles for relevant articles.

### Study selection

A reviewer screened titles and abstracts of all articles retrieved from the search before reviewing the full text of potentially eligible studies. A second reviewer performed independent full-text checks on a random subset (30%) of the studies, with 100% agreement.

### Data extraction

Two authors independently extracted data from the relevant studies. Data were then compared, and inconsistencies were resolved in consensus meetings. For all studies, the data extraction included first author, year, journal, title, cohort, country, number of MZ and DZ participants, MZ and DZ twin correlations with 95% confidence interval (CI), proportion of males and females, age at assessment, reporter (self-reported, parents or other informant, combined), child victimization type(s) assessed, and age at exposure. We also extracted information on study quality based on selected items from the Newcastle-Ottawa Scale: the population representativeness of the samples (whether the sample was broadly population representative and included >60% of the targeted or baseline sampling frame) and the validation of the childhood victimization measures (i.e., whether the measure was validated in previous studies).

### Statistical analysis

All analyses were performed in R version 4.1.1 (R Foundation). Datasets and scripts are available at: https://github.com/andrea-danese/victim_herit.git.

#### The classical twin design

We synthesized data on twin correlations from the selected studies based on the twin design, which compares correlations in a phenotype (childhood victimization in our case) between MZ and DZ twin pairs to estimate the extent to which individual differences in the phenotype are explained by genetic factors, shared environmental factors, and non-shared environmental factors [23]. Because MZ twin pairs are genetically identical, they are assumed to have a perfect correlation for latent additive genetic effects (A), whereas DZ pairs share on average 50% of their segregating genes and are thus assumed to have a correlation of 0.5 for A. In terms of the shared (family) environment (C), both MZ and DZ pairs are assumed to have a perfect correlation if reared together. The environmental factor that entails non-shared environmental effects (E) is uncorrelated across twins in MZ and DZ pairs and captures differences among twins within the same family, for example, effects due to peer relationships (as well as measurement error). The ratio of the MZ to DZ correlations indicates the extent to which additive genetic, shared environmental, or non-shared environmental influences account for variation in victimization. A 2:1 ratio indicates that shared influences are genetic rather than environmental, whereas a 1:1 ratio indicates that shared influences are environmental rather than genetic. A combination of genetic and shared environmental effects is indicated for any ratio in-between, i.e., if the DZ twin correlation is more than half that of the MZ twin pairs [26, 27]. Normally the models for the expected correlations in MZ and DZ pairs (A + C and 0.5 A + C, respectively) are fitted to the observed raw data to estimate the most likely estimates for A, C, and E.

#### Meta-analysis of ACE parameter estimation

The input for these analyses were MZ and DZ correlation matrices from the selected studies, with their associated number of twin pairs (sample sizes) used as weights.

Structural Equation Model (SEM)-fitting analyses were conducted in the R-based *OpenMx* package [28] to ascertain maximum likelihood estimates of the ACE parameters. Since the variance of the input correlation matrices is equal to one, a constraint was added to the model (A + C + E = 1) to correct for the degrees of freedom. Therefore, we effectively estimated only 2 parameters since the third one can be derived from knowing the other two. This constraint also means that we are estimating standardized parameter estimates. The precision of the ACE estimates is given by maximum likelihood 95% confidence intervals.

For the main analyses, we fit a model where all studies have a set of free ACE parameters. We then used multiple group analyses to cluster studies with the same characteristics and test if estimates of heritability vary between these groups. The different characteristics of the studies that we looked at were: the type of reporter (self-reported or reported by informants [including parents, teachers, peers]), types of victimization (child maltreatment [including physical abuse, sexual abuse, emotional abuse, physical neglect, and emotional neglect], or bullying), and age at exposure to victimization (in childhood [birth to 11 years], or adolescence [12–18 years]). To test between-subgroups equality across meta-analytical estimates, we performed a likelihood-ratio test on the ACE parameters. The test constrains the parameters across two subgroups and interprets the change in fit to examine whether using a single set of ACE parameters for both subgroups would lead to a significantly worse explanation of the observed correlations. Since the models are nested, their relative fit is established by the difference in chi-square (with df = 2).

#### Multi-level meta-analysis

We prioritized the meta-analysis of ACE parameters through the SEM approach described above because it provides a single overall model for all three parameters (e.g., ensuring the sum of the parameters equaled 1) and an overall test of differences in parameters’ estimates in subgroup analyses while accounting for the hierarchical nature of the data (with multiple effect sizes for study and cohort). However, the SEM approach did not include terms for sampling error and between-study heterogeneity, and could not provide estimations of heterogeneity, publication bias, or unduly effects of large studies.

As a sensitivity analysis for the SEM methods above, we therefore also ran three multi-level meta-analyses (one for each of the ACE parameters) with the *metafor* package in R. The inputs for the multi-level meta-analyses were the SEM-estimated ACE parameters for each study (see above). To account for the hierarchical nature of the data, we specified three levels of variance in effect sizes: random-sampling variance, within-study variance, and between-study variance. To estimate heterogeneity, we used the *I*^2^ statistic, which reflects the proportion of the observed variance that is due to true variation in effect sizes if sampling error was eliminated. To test for publication bias, we used an extension of Egger’s test for multi-level meta-analysis models [29], which tests for whether the study variance moderates the meta-analytic effect size. To test for undue influence of individual cohorts, we ran leave-one-out analyses by examining changes in estimates across permutations which omitted each cohort in turn. Finally, to test the impact of study quality on the findings, we undertook meta-regression analyses of heritability estimates to test the possible moderation effects by two key variables that were both informative and variable within the set of studies identified: the population representativeness of the samples and the validation of the childhood victimization measures.

## Results

### Search results

**Fig. 1:**
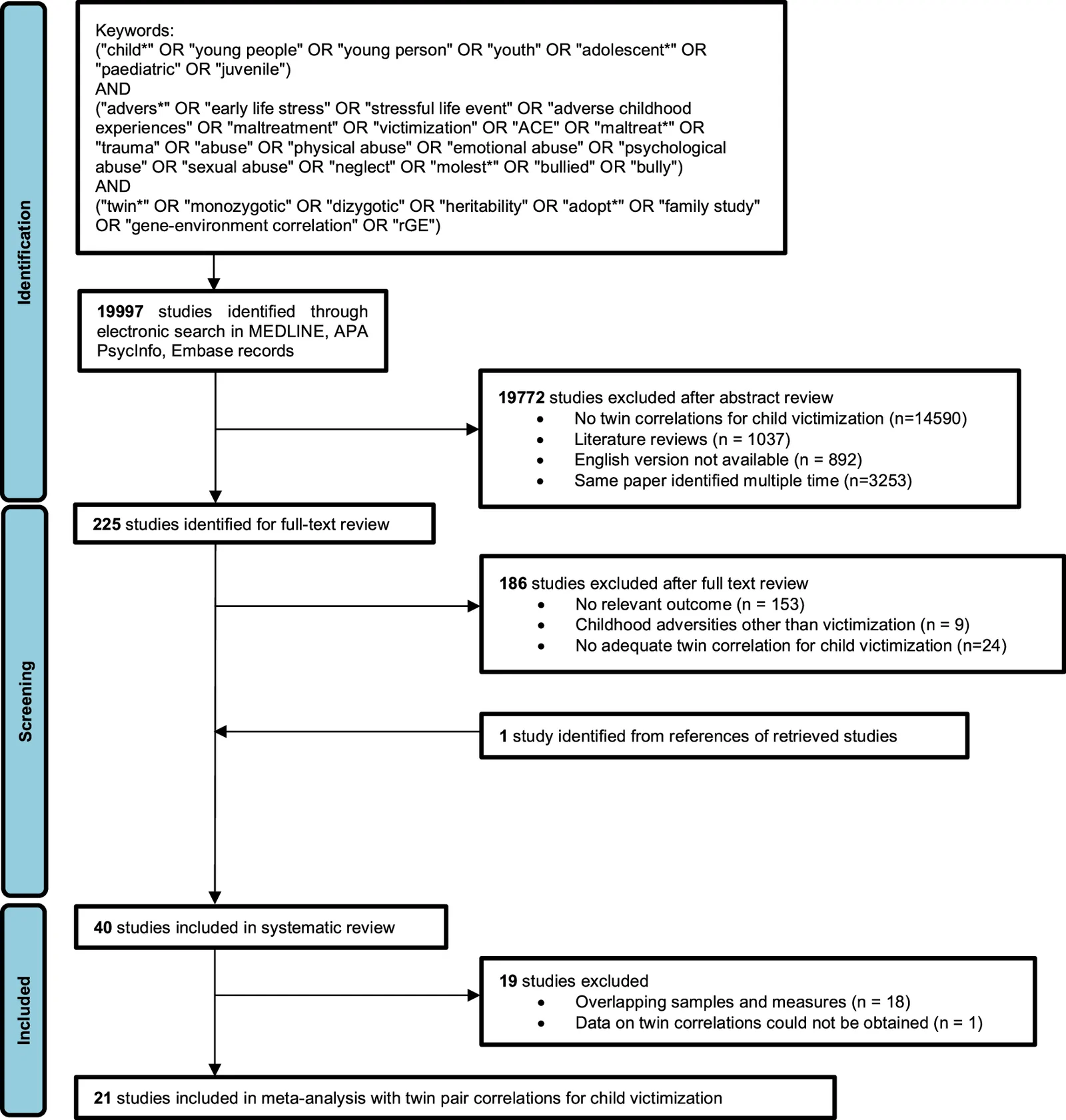
Study selection. PRISMA flowchart depicting study selection.

**Fig. 2:**
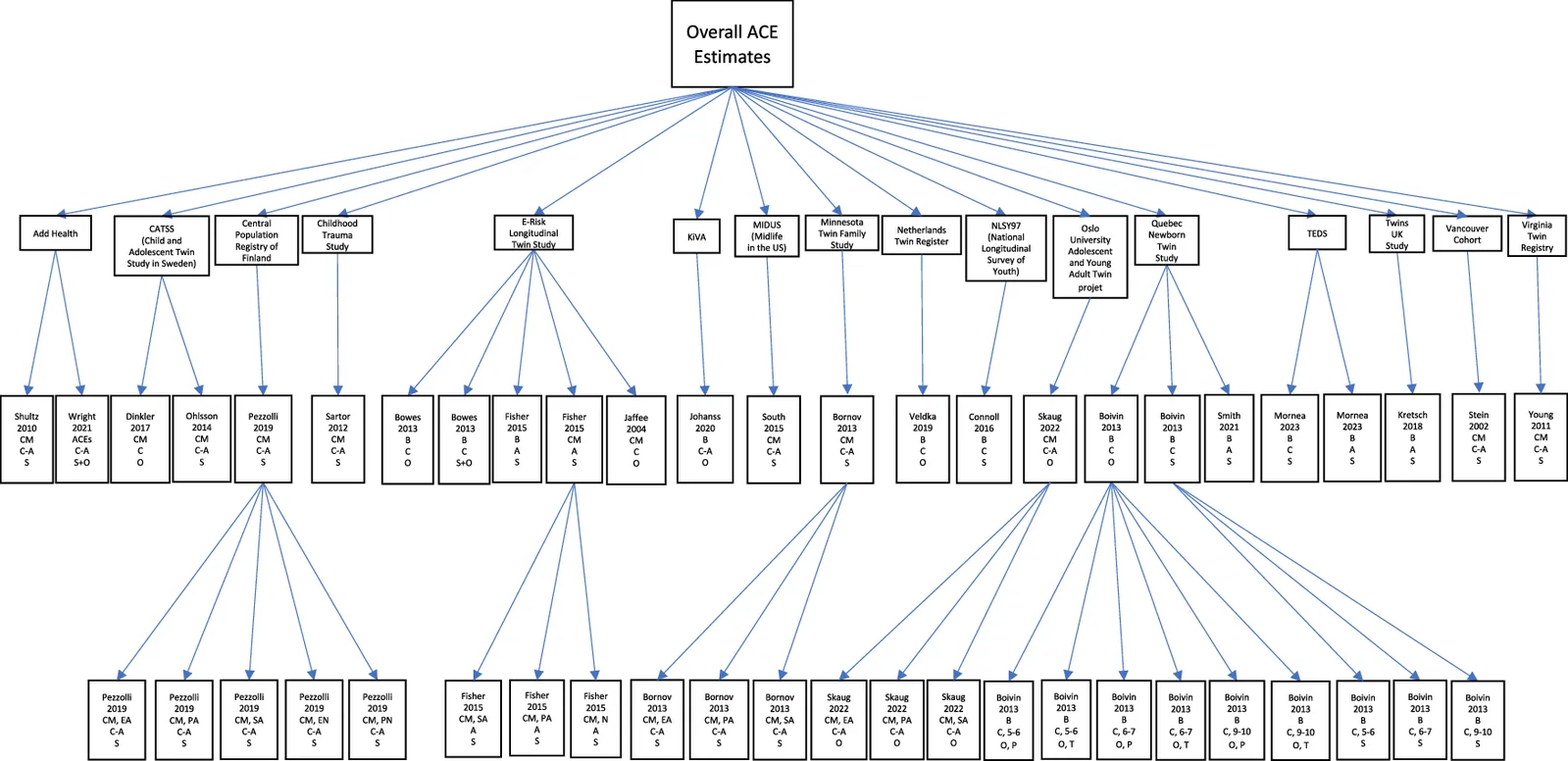
Structure of the data. Panels display information on the included studies related to victimization type (B bullying, CM child maltreatment) and subtypes (EA emotional abuse, EN emotional neglect, N neglect [emotional and physical], PA physical abuse, PN physical neglect, SA sexual abuse), age at exposure (A adolescence, C childhood [age in years if specified], C-A childhood and adolescence), and reporter type (O others, P peers, T teachers, S self, S + O = self and others).

The study selection process is summarized in Fig. 1. The characteristics of the studies included in the analysis are described in Table 1 (for full details, see https://github.com/andrea-danese/victim_herit.git). We identified *k* = 21 studies [30–50] with 42 effect sizes from 16 cohorts including 24,080 MZ and 38,714 DZ twins with relevant data. The structure of the available data is displayed in Fig. 2. Of the included studies, 10 (48%) were undertaken in samples that were broadly population representative and 14 (67%) used validated measures to assess childhood victimization (see Table S1 for details).

**Table 1:** Study characteristics.

Study	Cohort, Country	MZ/DZ(n)	Age at report (mean and/or range, years)	Reporter	Victimization type	Age at exposure (years)
[30]	Add Health, USA	612/894	23	Self	Child maltreatment	0–12
[31]	Add Health, USA	434/370	12–32	Combined self + parental reports	Child maltreatment (as part of a measure of Adverse Childhood Experiences)	0–18
[32]	Child and Adolescent Twin Study in Sweden (CATSS), Sweden	3578/9474	9	Parents	Child maltreatment	0–9
[33]	Child and Adolescent Twin Study in Sweden (CATSS), Sweden	1536/2914	18	Self	Sexual abuse	0–18
[34]	Central Population Registry of Finland, Finland	3248/6314	29 (18–49)	Self	Emotional abuse, physical abuse, sexual abuse, emotional neglect, physical neglect	0–18
[35]	Childhood Trauma Study, Australia	282/322	37	Self	Child maltreatment (as part of a measure of high-risk trauma)	0–18
[36]	E-Risk Longitudinal Twin Study (E-Risk), UK	1228/1004	7–12	Combined self + parental reports	Bullying	5–12
[37]	E-Risk Longitudinal Twin Study (E-Risk), UK	1132/927	18	Self	Bullying, sexual abuse, physical abuse, neglect	12–18
[38]	E-Risk Longitudinal Twin Study (E-Risk), UK	1228/1004	5	Parents	Physical abuse	0–5
[39]	KiVa, Finland	107/340	7–15	Peers	Bullying	7–15
[40]	Midlife in the United States (MIDUS), USA	700/644	45 (25–74)	Self	Child maltreatment	0–18
[41]	Minnesota Twin Family Study, USA	1792/972	20–29	Self	Emotional abuse, physical abuse, sexual abuse	0–18
[42]	Netherlands Twin Register, Netherlands	3338/5784	9 (7–13)	Teachers	Bullying	5–12
[43]	National Longitudinal Survey of Youth (NLSY97), Finland	54/448	14 (12–16)	Self	Bullying	5–12
[44]	Oslo University Adolescent and Young Adult Twin Project, Norway	1080/1688	19 (18–23)	Self	Emotional abuse, physical abuse, sexual abuse	0–18
[45]	Quebec Newborn Twin Study, Canada	393/544	6–10	Peers, teachers, self	Bullying	5–10
[46]	Quebec Newborn Twin Study, Canada	314/492	13–17	Self	Bullying	12–17
[47]	Twins Early Development Study (TEDS), UK	4208/7420	12 and 16	Self	Bullying	11–16
[48]	Twins UK Study, UK	1296/1214	60	Self	Bullying	12–18
[49]	Vancouver cohort, Canada	444/368	34 (16–86)	Self	Child maltreatment (as part of a measure of assaultive trauma)	0–18
[50]	Virginia Twin Registry, USA	1720/1284	35 (19–56)	Self	Child maltreatment	0–15

When multiple reporters are listed, they provide independent measures unless described otherwise (combined reports).

### Overall A, C, and E parameter estimates

**Fig. 3:**
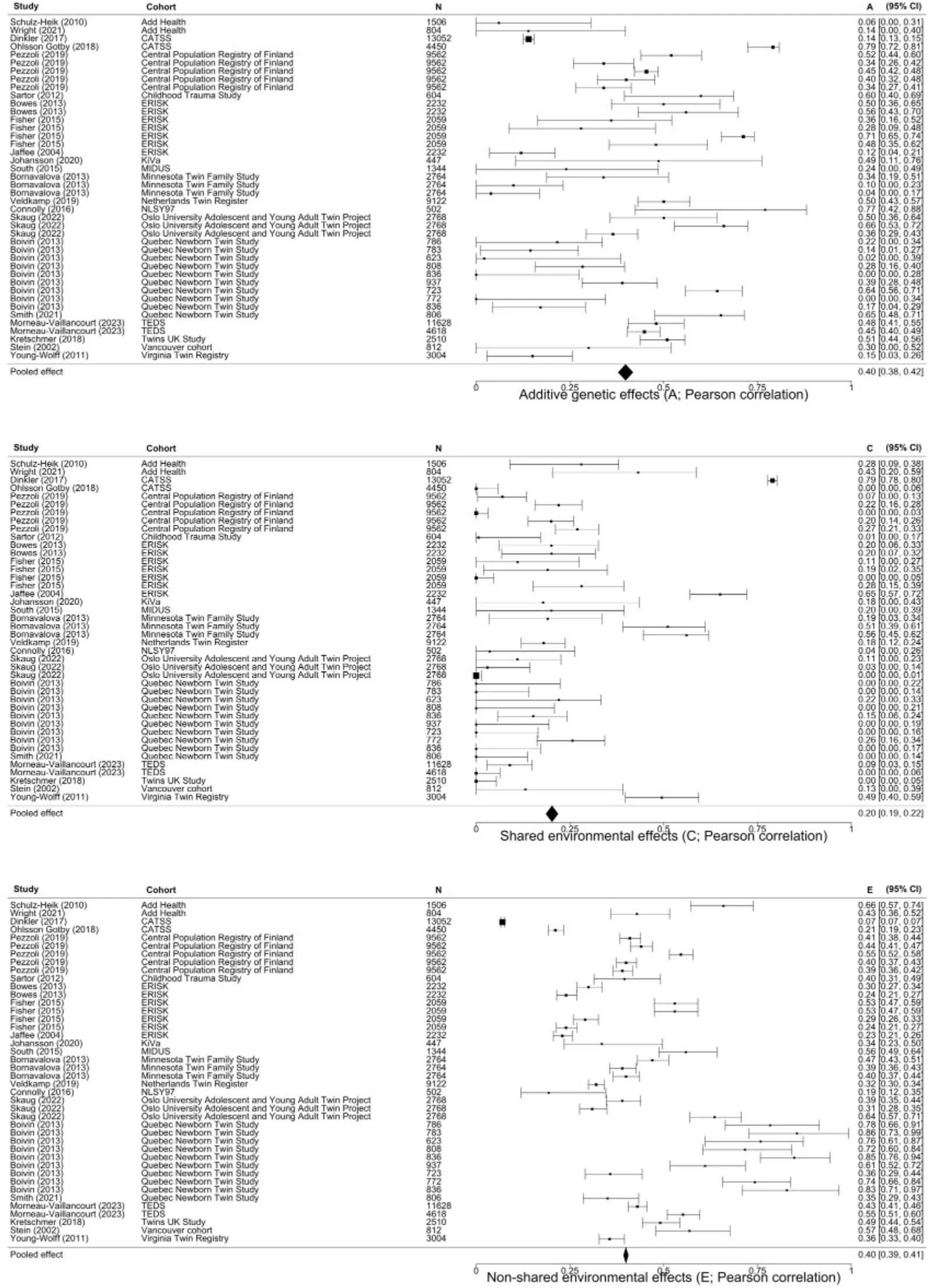
Forest plots of ACE parameter estimates for child victimization based on Structural Equation Model-fitting.

**Fig. 4:**
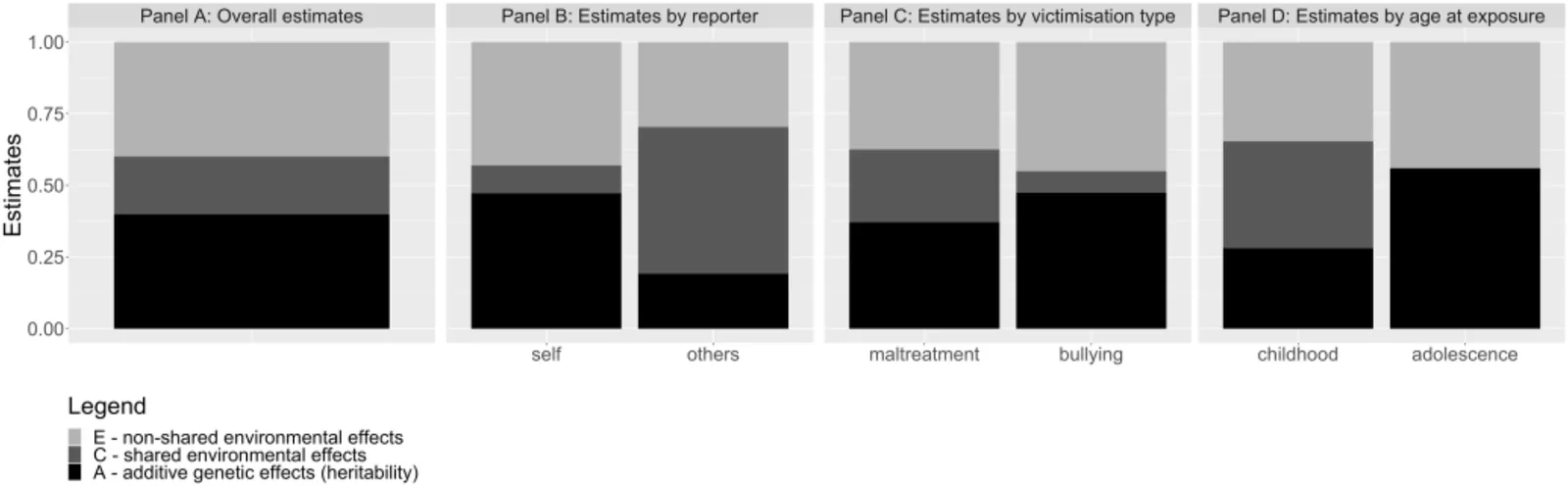
Genetic and environmental influences on childood victimization. The bar plot summarizes the metaanalytical results of ACE parameter estimates for child victimization in the overall analyses (**A**) and subgroup analyses (**B**–**D**) based on Structural Equation Model-fitting.

A meta-analysis using SEM showed that the overall parameter estimates for influences on child victimization were 0.40, 95% CI = 0.38–0.42 for genetic influences (A; heritability); 0.20, 95% CI = 0.19–0.22 for shared environmental influences (C); and 0.40, 95% CI = 0.39–0.41 for non-shared environmental influences (E), as displayed in Fig. 3 and in Fig. 4A.

In sensitivity analyses, a multi-level meta-analysis of the estimated additive genetic influences showed similar estimates of A = 0.41, 95% CI = 0.31–0.50, with heterogeneity of I^2^ = 98.95%. The Egger’s test was statistically non-significant (p = 0.075), suggesting no clear evidence of publication bias. Leave-one-out analyses showed estimates ranging between 0.39 and 0.43 with overlapping confidence intervals, suggesting no undue influence of individual cohorts. A multi-level meta-analysis of the estimated shared environmental influences showed similar estimates of C = 0.23, 95% CI = 0.12–0.34, with heterogeneity of I^2^ = 99.03%, no evidence of publication bias (Egger’s test p = 0.36), and leave-one-out analyses ranging between 0.19 and 0.25. Finally, a multi-level meta-analysis of the estimated non-shared environmental influences showed similar estimates of E = 0.43, 95% CI = 0.35–0.51, with heterogeneity of I^2^ = 98.83%, no evidence of publication bias (Egger’s test p = 0.66), and leave-one-out analyses ranging between 0.41 and 0.45. In meta-regression analyses, neither sample representativeness nor measurement validation explained the heterogeneity in heritability estimates (A = 0.47, 95% CI = 0.34–0.58 in 11 studies with non-representative samples and A = 0.35, 95% CI = 0.22–0.47 in 10 studies with representative samples, with moderation p-value = 0.1796; A = 0.43, 95% CI = 0.23–0.60 in 7 studies with non-validated measures of childhood victimization and A = 0.40, 95% CI = 0.29–0.50 in 14 studies with validated measures, with moderation p-value = 0.7719). Based on a-priori hypotheses and in light of the high heterogeneity estimate, we then ran targeted subgroup analyses.

### Differences in parameter estimates by reporter

A meta-analysis for self-reported measures of child victimization was based on 15 studies (29 twin correlations) and yielded parameter estimates of A = 0.47 (95% CI = 0.45–0.50), C = 0.10 (95% CI 0.08–0.12), and E = 0.43 (95% CI 0.42–0.44). A meta-analysis for informant-based measures of child victimization was based on 6 studies (11 twin correlations), and yielded estimates of A = 0.19 (95% CI = 0.16–0.22), C = 0.51 (95% CI = 0.49–0.54), and E = 0.30 (95% CI = 0.29–0.31). A likelihood-ratio test showed that parameter estimates for self-reported measures versus informant-reported measures were different, with Χ^2^(df = 2) = 1423.6, p < 0.00001. Compared to informant-reported victimization, self-reported victimization involved greater genetic and non-shared environmental influences, but lower shared environmental influence (Fig. 4B).

### Differences in parameter estimates by victimization type

A meta-analysis for child maltreatment was based on 11 studies (19 twin correlations) and yielded estimates of A = 0.37 (95% CI = 0.35–0.39), C = 0.25 (95% CI = 0.24–0.27), and E = 0.38 (95% CI = 0.37–0.38). A meta-analysis for bullying was based on 9 studies (19 twin correlations) and yielded estimates of A = 0.47 (95% CI = 0.43–0.51), C = 0.08 (95% CI = 0.04–0.11), and E = 0.45 (95% CI = 0.44–0.46). A likelihood-ratio test showed that parameter estimates for maltreatment versus bullying were different, with Χ^2^ (df = 2) = 341.2, p < 0.00001. Compared to bullying, child maltreatment involved lower genetic and non-shared environmental influences, but higher shared environmental influence (Fig. 4C).

### Differences in parameter estimates by age at exposure

A meta-analysis for exposure to victimization in childhood (0-11 years) was based on 7 studies (16 twin correlations) and yielded estimates of A = 0.28 (95% CI = 0.25–0.31), C = 0.37 (95% CI = 0.35–0.40), and E = 0.44 (95% CI = 0.42–0.46). A meta-analysis for exposure to victimization in adolescence (12–18 years) was based on 4 studies (7 twin correlations) and yielded estimates on A = 0.56 (95% CI = 0.54–0.58), C = 0.00 (95% CI = 0.00–0.03), and E = 0.44 (95% CI = 0.42–0.46). A likelihood-ratio test showed that parameter estimates for victimization in childhood versus adolescence were different, with Χ^2^ (df = 2) = 420.8, p < 0.00001. Compared to adolescent victimization, childhood victimization involved lower genetic influence, but higher shared environmental influence (Fig. 4D).

## Discussion

We have examined the genetic and environmental influences on childhood victimization in a meta-analysis of k = 21 studies from 16 cohorts with 62,794 participants. We found that heritability (additive genetic influences) accounted for 40% of the variance for childhood victimization, shared environmental influences for 20%, and non-shared environmental influences for 40%. The findings were consistent across estimation methods. There was no clear evidence of publication bias or undue influence of large studies. Furthermore, the quality of the studies included (indexed by the population representativeness of the samples and the validation of the childhood victimization measures) did not significantly influence the findings. We found that genetic and environmental influences varied based on the reporter for victimization, the type of victimization, and the age at victimization.

The 40% heritability estimate for childhood victimization is in line, for example, with moderate heritability estimates for other interpersonal experiences like social interactions (32%; including community life, basic interpersonal interactions, complex interpersonal interactions, family relationships, formal relationships, informal social relationships, intimate relationships) [15], trauma (36%) [20], and negative life events (39%) [20]. Our findings can also be triangulated across other genetically informative methods. For example, adoption studies have shown that adoptees’ birth parents’ characteristics are associated with harsh parenting by adoptive parents [51]; extended family design studies [52] and genome-wide association studies (GWAS) [53, 54] have shown that maltreatment is partly heritable; and polygenic score studies have shown that genetic liabilities to mental health problems are associated with maltreatment [55] and bullying [56]. Of note, heritability estimates varied significantly in our subgroup analyses: the heritability of childhood victimization was greater for self-reports (47%) than informant-based measures (19%); for victimization by peers (bullying, 47%) than by adults (maltreatment, 37%); and for victimization in adolescence (56%) than in childhood (28%). These differences provide some insights into how genetic influences may affect childhood victimization risk.

Genetic influences may increase the likelihood of exposure to childhood victimization through gene-environment correlations (rGE) [57]. For example, cognitive difficulties are partly heritable, and parents with cognitive difficulties may find it harder to provide adequate care for their children and also pass these difficulties on to their children (passive rGE) [8]; neurodevelopmental conditions are partly heritable, and children with neurodevelopmental conditions might be more likely to exhibit behavioral difficulties, which could trigger maladaptive parental practices (evocative rGE) [58]; and personality is partly heritable, and children with higher neurotic personality might be more likely to become more socially isolated and vulnerable to bullying victimization (active rGE) [59]. Our subgroup analyses highlight that heritable influences are stronger for victimization experiences in adolescence (vs childhood) and by peers (vs adults), when evocative and active rGE might be more likely to take place, consistent with evidence from large SNP-based analyses [53] and polygenic score studies [60, 61].

Heritable influences may also increase the likelihood that certain childhood experiences are appraised, remembered, and/or reported as victimization [62]. Previous research from our team showed that prospective measures of victimization typically based on informant reports and retrospective measures based on self-reports identify largely non-overlapping groups [19], and that self-reported measures are more strongly associated with psychopathology [63–65]. Our subgroup analyses here highlighted that heritable influences are stronger for self-reported vs informant-reported measures of childhood victimization. The finding provides evidence of etiological differences between the constructs identified by two measures, consistent with findings from a systematic review showing greater heritability for self-reported environmental experiences versus more objective observer ratings [20]. Heritable influences may affect appraisal as well as the formation and/or retrieval of memories of childhood victimization. For example, polygenic scores for Autistic Spectrum Disorder (ASD) are associated with self-reports of childhood victimization [53, 66–68], and individuals with ASD may be more likely to interpret life experiences as victimization than neurotypical peers and, thus, form relevant memories [69]. Furthermore, executive functions are partly heritable and may contribute to the successful suppression of unwanted memories and forgetting over time (i.e., suppression-induced forgetting) [70]; as such, individuals with lower executive functions might be more likely to self-report childhood experiences.

Environmental influences on childhood victimization are more commonly considered. Our analyses add to the existing literature by discriminating between shared and non-shared environmental influences, namely environmental factors that make children growing up in the same family similar or dissimilar, respectively [71]. Shared and non-shared environmental influences combined explain a larger proportion of variance in childhood victimization than heritable influences, but it is the relative contribution of these influences that could provide novel etiological insights. Overall, shared environmental influences on childhood victimization are weaker than non-shared environmental influences, consistent with findings across several human traits [15]. Shared environmental influences capture the family environment (“nurture”) and may reflect the contribution of known risk factors for childhood victimization, such as family conflict, poverty, or parental mental illness [72]. In our analyses, shared environmental influences appear to have stronger effects on maltreatment than bullying and in childhood compared to adolescence, reflecting the greater relevance of family units for maltreatment and during childhood. Shared environmental influences may also reflect measurement features in child victimization research, as they appear to be stronger for informant reports (typically by parents) than self-reports, which could reflect parents answering in a similar manner for both twins. Non-shared environmental influences point to effects that are unique to different children even within the same family, including differential treatment within the family, differences in peer groups or school experiences, or accidents. In our analyses, non-shared environmental influences appear to have stronger effects on bullying than maltreatment, and on victimization in adolescence relative to childhood, reflecting the relevance of extra-familiar experiences during adolescence. Non-shared environmental influences may also reflect other measurement features in childhood victimization research (e.g., imperfect test-retest reliability), as they appear to be larger for self-reported measures.

Our findings should be interpreted in the context of some limitations. First, the twin analyses are based on certain assumptions [23] that have only been partly addressed [73, 74]. In addition, the twin designs might present challenges to estimating the heritability for child maltreatment, because it is likely that if one twin is maltreated, so too is the other twin within the same family, restricting the variance to be decomposed. The ACE estimates from the classical twin design examined here may also differ from estimates from extended twin designs that directly model gene-environment interplay [75]. However, findings from our meta-analysis of twin studies, such as the presence of heritable influences on childhood victimization, can be triangulated across other genetically informative designs, as described above.

Second, the ACE parameter estimates from the multi-level meta-analyses show very high levels of heterogeneity, which suggests that the estimates should be interpreted cautiously. We found that key variables, such as the reporter for victimization, the type of victimization, and the age at victimization can at least partly explain this heterogeneity. However, we note that the number of studies in some of the subgroup analyses was small (e.g., analyses by age at exposure). In addition, because of the limited information in the original studies, we were not able to look at other variables that might have contributed to the observed heterogeneity in parameter estimates, for example, systematic variation based on definitions of maltreatment or bullying, focus on different subtypes of maltreatment or bullying, presence of current mental health at the time of reporting, or personality [62]. This variable should be further explored in future studies.

Third, findings might not be broadly generalizable. Because all twin studies identified were undertaken in high-income settings, such as the USA, UK, Scandinavian countries, Canada, and Australia, the variance in environmental influences is likely restricted. This is important because ACE estimates are not constant but can vary depending on context. For example, ACE estimates can vary in different countries [15], and a restricted geographical range might bias the overall results. ACE estimates can also change over time with shifting lifestyles and cultural/societal norms [76], and studies in different eras might lead to different results. Furthermore, the victimization experiences observed in general population samples or convenience samples included in the meta-analysis may not be representative of the more extreme victimization experiences observed in disadvantaged portions of the population, which are typically less likely to take part in research and should be more actively investigated in future studies. Therefore, future studies should sample from more diverse environments including low-resource settings. There are also other types of childhood victimization that, because of the limited number of relevant studies or methodological challenges in applying twin models (e.g., dating violence, community violence), have not been directly examined in this paper and might show different ACE estimates compared to the types of victimization examined here.

Fourth, because childhood victimization was predominantly reported by parents while adolescent victimization was predominantly self-reported, it is possible that subgroup analyses by age at exposure could at least partly reflect reporting effects. Fifth, the analyses could not discriminate between types of rGE or influences on appraisal or memory of victimization experiences, which should be further investigated. Despite these potential limitations, our findings have implications for research, clinical practice, and public health.

Researchers can build on these findings to enrich ecological models of childhood victimization [77, 78]. The hypotheses outlined above about the potential genetic and environmental mechanisms underlying risk for childhood victimization need to be directly tested within genetically sensitive and causal frameworks [79, 80]. This research will provide novel mechanistic insights into potentially modifiable targets for preventative interventions. The findings also highlight etiological differences across measures of childhood victimization (by reported, type, or age) currently conflated in a single construct. Such measurement heterogeneity can be clinically meaningful [63–65, 81] and should be further investigated in future research.

Clinicians can capitalize on these findings to develop more comprehensive risk formulation and management plans. Child safeguarding is often based on simplistic models (e.g., the ‘toxic trio’ [82]) that are limited in scope and utility. Our results suggest that better detection and mitigation of victimization risk will come from a more granular understanding of the reciprocal relationships between characteristics of children, families, and their broader social environment. Furthermore, the findings of heritable influences on child victimization add to the evidence that victimization can be associated with mental health and cognitive outcomes through both causal and non-causal mechanisms [8, 83]. In particular, clinicians should consider that observed associations between child victimization and clinical outcomes may at times emerge from shared genetic liability [84]. Such alternative formulations may provide new modifiable targets to prevent the recurrence of childhood victimization.

Policymakers can play a key role in public understanding and mitigation of the risk for childhood victimization. Evidence of heritable influences on victimization should not be interpreted to suggest that exposure to victimization is genetically determined, is immutable, or is the child’s fault. The findings similarly do not imply that changes in victimization risk can only be, or should be, achieved through interventions targeting children or their families. For example, genetic liability for higher BMI or autism predicts greater victimization risk [56, 66], but reduction in victimization risk might be achieved by changing societal norms about body image or neurodiversity (e.g., reducing fat-shaming or de-stigmatizing different ways of thinking, learning, and behaving).

## Supplementary information

Online supplement

PRISMA checklist
