# Supplementary material for: Genetic and environment influences on childhood victimization: a systematic review and meta-analysis
Source: Mol Psychiatry. 2024 Dec 11;30(5):2228–38. doi: 10.1038/s41380-024-02868-z (PMC12014478; doi:10.1038/s41380-024-02868-z)
Supplement: Supplementary file 1 — Online supplement [file 41380_2024_2868_MOESM1_ESM.docx]

# **Genetic and environment influences on childhood victimization: a systematic review and meta-analysis**

Tarik Dahoun, Alicia Peel, Jessie Baldwin, Oonagh Coleman, Stephanie J. Lewis, Jasmin Wertz, Frühling Rijsdijk, Andrea Danese

**Deviations from pre-registered protocol**

We want to highlight two deviations from our registered protocol.

Search results. In our registered protocol, we were intentionally over-inclusive regarding the search terms to ensure that we could identify all studies related to genetic and environmental influences on childhood victimization. In particular, we included search terms to identify SNP-based heritability studies, extended family design studies, and adoption studies. As a result, in addition to classical twin design studies, we also identified 3 studies from 2 cohorts focusing SNP-based heritability (Bolhuis et al, 2021, Eur Child Adolesc Psychiatry <https://link.springer.com/article/10.1007/s00787-021-01727-4>; Dalvie et al, 2020, Transl Psychiatry: <https://www.nature.com/articles/s41398-020-0706-0>; Warrier et al, 2021, Lancet Psychiatry: <https://www.thelancet.com/journals/lanpsy/article/PIIS2215-0366(20)30569-1/fulltext>); 3 studies from two cohorts on extended family designs (Pittner, 2019, Dev Psychopathol: <https://www.cambridge.org/core/journals/development-and-psychopathology/article/abs/genetic-and-environmental-etiology-of-child-maltreatment-in-a-parentbased-extended-family-design/09BB06134CE8DFC11FCD3B20A4B15D74>; Pittner, 2020, Child Maltreatment: <https://journals.sagepub.com/doi/full/10.1177/1077559519888587>; Warrier et al, 2021, Lancet Psychiatry: <https://www.thelancet.com/journals/lanpsy/article/PIIS2215-0366(20)30569-1/fulltext>); but no adoption studies. Because of the very low number of studies with alternative designs identified, we only pursued a quantitative summary (meta-analysis) for classical twin design studies.

Quality assessment. In our registered protocol, we had planned to examine study quality through the Newcastle-Ottawa Scale (NOS) for observational / cohort studies. However, it became clear that this often-used instrument was not appropriate for our specific circumstances: for example, non-exposed participants were selected from the same population (the same family) as exposed participants; comparability of exposed/unexposed participants was irrelevant our analyses did not focus on associations between exposure and outcome but on decomposition of univariate ACE parameters. As such, the full NOS was unable to discriminate quality across the studies identified. Therefore, we have extracted two key variables that are both informative and varying within the set of studies identified: the population representativeness of the samples (whether the sample was broadly population representative and included >60% of the targeted or baseline sampling frame) and the validation of the childhood victimization measures (i.e., whether the measure was validated in previous studies). Details of this quality assessment exercise are reported below.

**Table S1.** Quality assessment

| **Study** | **Representativeness of the sample** | **Validation of the child victimization measure** | |
| --- | --- | --- | --- |
|  | **Score** | **Score** | **Measure details** |
| Boivin 2013 | 1 | 1 | Peer nomination system |
| Bornovalova 2015 | 1 | 1 | Trauma Assessment for Adults (TAA), Childhood Experiences Questionnaire (CEQ) |
| Bowes 2013 | 1 | 1 | Interview with children and parents |
| Connolly 2016 | 1 | 0 | One question on bullying victimization |
| Dinkler 2017 | 1 | 1 | Life Stressor Checklist-Revised (LSC-R) |
| Fisher 2015 | 1 | 1 | Juvenile Victimization Questionnaire (JVQ) |
| Jaffee 2004 | 1 | 1 | Interview with mothers (from the Multi-Site Child Development Project) |
| Johansson 2020 | 1 | 1 | Participant Role Questionnaire (PRQ; peer nomination) |
| Kretschmer 2018 | 0 | 0 | One question on bullying victimization |
| Morneau-Vaillancourt 2023 | 0 | 1 | Multidimensional Peer Victimization Scale (MPVS) |
| Ohlsson Gotby 2018 | 0 | 1 | Life Stressor Checklist-Revised (LSC-R) |
| Pezzoli 2019 | 1 | 1 | Childhood Trauma Questionnaire Short Form (CTQ-SF) |
| Sartor 2012 | 0 | 0 | Trauma checklist: ‘high risk' trauma includes maltreatment experiences |
| Schulz-Heik 2010 | 1 | 0 | Four questions on maltreatment |
| Skaug 2022 | 0 | 1 | Childhood Trauma Interview (CTI) |
| Smith 2021 | 0 | 1 | Social experiences questionnaire (SEQ) |
| South 2015 | 0 | 1 | Conflict Tactics Scale (CTS) |
| Stein 2002 | 0 | 0 | Trauma checklist: 'assaultive trauma' includes maltreatment experiences |
| Veldkamp 2019 | 0 | 0 | Four questions on bullying victimization |
| Wright 2021 | 0 | 1 | ACE questionnaire includes maltreatment experiences |
| Young-Wolff 2011 | 0 | 0 | Three questions on maltreatment |
